# A Case of Complete Inversion of the Uterus Reduced

**Published:** 1872-04-15

**Authors:** E. R. Willard

**Affiliations:** Wilmington, Ill.


### A CASE OF COMPLETE INVERSION OF THE UTERUS REDUCED.

BY E. R. WILLARD, M.D., WILMINGTON, ILL.

Inversion of the uterus is of exceeding rare occurrence, in so much that an average of only one case is reported out of nearly a hundred thousand births in some of the best lying-in hospitals on the Eastern continent; and many accoucheurs in extensive obstetrical practice for a long series of years, have never met with a single case. In fact it is a mal-position but seldom if ever diagnosed two hundred years ago. Since that time, however, its symptoms, signs, pathology and great danger have been thoroughly studied and fully appreciated by the profession; and, although such is the case, we find the displacement to have been frequently mistaken for polypus, prolapsus, fibroid, or even to have remained undiscovered for a number of years, or until after death, by physicians of good reputation, and graduates from the best medical schools in the country. This we attribute not so much to the want of medical knowledge and skill, as from the exceeding rare occurrence of this difficulty, thereby throwing the physician completely off his guard—that is, causing him to form a diagnosis without a sufficiently thorough examination.

Systematic writers differ very much in their classification of the varieties of this affection. For the more practical purposes of pathology, diagnosis, prognosis and treatment, we would make the following heads:

| | | |
|---|---|---|
| 1st. Partial Inversion, | Acute, | Spontaneous. |
| | | Artificial. |
| | Chronic, | Spontaneous. |
| | | Artificial. |
| 2d. Complete Inversion, | Acute, | Spontaneous. |
| | | Artificial. |
| | Chronic, | Spontaneous. |
| | | Artificial. |

Many writers would still divide spontaneous and artificial into active and passive uterine inversion; but as that depends upon the degree of paralysis or inertia of the uterus in the former, and upon the amount of artificial force in the latter—that is, spontaneous passive inversion signifying spontaneous inversion from complete paralysis of the whole organ, and artificial passive inversion merely applies force to the same condition of the uterus to produce the inversion, while the term active is applied to the same kinds of inversion where only a part of the organ is paralyzed, thereby producing what is called hour-glass contractions in some cases—therefore we prefer the more general classification above.

Where the paralysis exists at the lower portion of the uterus, including the cervix, with active contractions of the fundus, together with positive bearing-down, we have frequently discovered partial temporary inversion, which would be immediately relieved so soon as active contractions of the lower portion of the uterus was secured. It is in this way, we think, a greater share of the cases of post-partem spontaneous inversion takes place. We are borne out in these conclusions, to some extent, in the fact that the accident seldom occurs in the hands of experienced practitioners, where these conditions are promptly met at the time.

That we always have partial or complete paralysis of the uterus in all cases of inversion, whether spontaneous or artificial, is now generally admitted by all writers upon that subject.

Another very important matter, in a medico-legal point of view, presents itself here, and that is the relative frequency of spontaneous inversion as compared with that of artificial inversion. For the purpose of presenting this matter clearly, in connection with the various causes, modes of treatment, and results, we have prepared the accompanying table, from which we find out of fifty-three cases taken promiscuously from the most reliable men in the country, commencing at the fifty-ninth case in the table, that twenty-eight were entirely spontaneous, while others were partially so, as no great amount of force was used to produce the displacement.

In this estimate we take only those cases of post-partem inversion, leaving out one case of fibro-cystis that is included among the spontaneous in the table from the fifty-ninth case.

As to treatment, it will be seen by reference to the same table that about one-third of all those who underwent the operation of amputation died; while of those who were treated by taxis for reduction, only about one-ninth died.—thus showing that by removing the organ, the fatality is as great as in the most malignant epidemic of cholera, or as in the operation of ovariotomy. We also find that the comparative length of time after the accident is as great, if not greater, in those cases treated by taxis, as in those by amputation: therefore it cannot be said that the former were acute cases, and consequently easily reduced.

From my limited experience, and the success claimed by Dr. Thomas for his method of drawing down the uterus and then forcing it up again, I am inclined to the opinion that a systematic course of manipulation, by periodic pressure of from three to five minutes, with a force of from ten to twenty pounds, with a period of rest of five minutes between, could be continued a greater number of hours, with less danger of producing inflammation, as the vitality of the tissues would not thereby be endangered as they always are under steady continuous pressure, and the relaxation would not be less speedy. As to the time recommended for continuing the manipulation at any one setting, I would be governed in that much the same as in a case of labor, and calculate that ten or twelve hours would not be more dangerous to the patient, provided the parts were not handled too roughly, than in natural parturition.

We are borne out in these conclusions from the accompanying cases reported, as that of Dr. White's is the only one where peritonitis and death supervened as the result of the manipulation necessary for reduction.

In the accompanying table we have only submitted a sufficient number of cases taken promiscuously, to form something like a correct comparison of results.

*Table of Cases in which the Uterus was Removed for Inversion. Also, Reduction of the Inverted Uterus, with Comparison of Results.*

| Number. | Years. | Age. | Mode of operating. Ligation. | Amputation. | Reduction. | Causes. Post Partum. | Polypus. | Fibroid. | Time after Accident. | Results of treatment. Successful. | Unsuccessful. | Kinds. Spontaneous. | Artificial. | Authority. | Remarks. |
|---|---|---|---|---|---|---|---|---|---|---|---|---|---|---|---|
| 1 | 1768 | | " | | | " | | | 17 days. | " | | | | Faiver. | Uterus was 27 days in separating. |
| 2 | 1824 | | " | | | " | | | 30 days. | " | | | | Rheineck. | Uterus came away in a few days. |
| 3 | 1818 | | " | " | | | " | | | " | | " | | Newnhan. | Uterus separated in seven days. |
| 4 | 1822 | | " | | | | " | | | " | | " | | Staub. | Knife used after the application of ligature. |
| 5 | 1835 | | " | | | " | | | | " | | | | Bouchet. | Ligature cut through on 14th day. |
| 6 | | | " | | | " | | | 3 years. | " | | | | Gooch. | Ligature came off in 14 days. |
| 7 | 1840 | | " | | | " | | | | " | | | | Harrison. | Uterus removed in 14 days. |
| 8 | 1836 | | " | | | " | | | | " | | | | Bloxam. | Uterus supposed to be polypus until after removal. |
| 9 | 1837 | | " | | | " | | | | " | | | | Kuttler. | Separated in three days. |
| 10 | 1838 | | " | | | | | | | " | | | | Williams. | |
| 11 | 1843 | | " | | | | | | 12 years. | " | | | | Esselman. | Ligated for a polypus; came off in 18 days. |
| 12 | 1846 | | " | | | " | | | | " | | | | Greyson. | Came off on 9th day. |
| 13 | 1852 | | " | | | " | | | 1 year. | " | | | | Betschler. | Came off on 14th day. |
| 14 | 1855 | | " | | | " | | | | " | | | | Oldham. | 22 days in separating. |
| 15 | 1861 | | " | | | | | | | " | | | | Courty. | Separated in 13 days. |
| 16 | 1863 | | " | | | | | | | " | | | | Dale. | Death from cancer in two months. |
| 17 | 1784 | | " | | | | " | | | | " | " | | Lammonier. | Death in one month after operation. |
| 18 | 1816 | | " | | | | " | | 3 months. | | " | " | | Peletin. | Death on fifth day. |
| 19 | 1864 | | " | | | " | | | | | " | | | Boyer. | Inversion mistaken for polypus; died in 12 days. |
| 20 | 1830 | | " | | | " | | | | | " | | | Symonds. | Inversion mistaken for polypus; died in 8 days. |
| 21 | 1852 | | " | | | " | | | 8 months. | | " | | | Deronbaix. | Death on 23d day. |
| 22 | 1855 | | " | | | " | | | 6 months. | | " | | | Coots. | Death on 16th day. |
| 23 | 1860 | | " | | | | | " | | | " | " | | Betschler. | Death on 24th day. |
| 24 | 1839 | | | " | | | " | | | " | | " | | Luytgarens. | Arteries ligated; healed in 10 days. |
| 25 | 1845 | | | " | | " | | | 13 months. | " | | | | Michalowski. | Recovered in 14 days. |
| 26 | 1678 | | | " | | " | | | | | " | | | Motte. | Death followed in a few days. |
| 27 | 1788 | | | " | | | | | | | " | | | Delayre. | Died in three days. |
| 28 | 1858 | | | " | | " | | | | | " | | | Aran. | Amputation by ecraseur; died in 59 hours. |

| | | | Mode of operating. | | | Causes. | | | | Results of treatment. | | Kinds. | | | |
|---|---|---|---|---|---|---|---|---|---|---|---|---|---|---|---|
| Number. | Years. | Age. | Ligation. | Amputation. | Reduction. | Post Partum. | Polypus. | Fibroid. | Time after Accident | Successful. | Unsuccessful. | Spontaneous. | Artificial. | Authority. | Remarks. |
| 29 | 1859 | | | " | | " | | | 12 months. | | " | | | McClintock. | By ecraseur; died in 59 hours. |
| 30 | 1864 | | | " | | | " | | | | " | " | | Wilson. | By ecraseur. |
| 31 | 1861 | | | " | | " | | | 7 months. | | " | | | Veit. | By ecraseur. |
| 32 | 1670 | | " | " | | | | | | " | | | | Vicuseium. | Ligature and knife both used. |
| 33 | 1787 | | " | " | | | " | | | " | | " | | Desault and Bodeloque. | |
| 34 | 1802 | | " | " | | | | | | " | | | | Hunter. | |
| 35 | 1804 | | " | " | | | | | | " | | | | Chevalier. | This case is reported as successful, although the patient died in a few days after the operation. |
| 36 | 1806 | | " | " | | | " | | | " | | | | Clarke. | Uterus supposed to be a polypus, and amputated for such. |
| 37 | 1811 | | " | " | | " | | | 5 weeks. | " | | " | | Baxton. | |
| 38 | 1818 | | " | " | | " | | | 18 months. | " | | " | | Windsor. | Amputation 12 days after ligation; healed in 75 days. |
| 39 | 1820 | | " | " | | | " | | | " | | | | Bœttger. | Ligation after amputation to stop hemorrhage. |
| 40 | 1821 | | " | " | | | " | | | " | | | | Weber. | Recovered in four weeks. |
| 41 | 1831 | | " | " | | " | | | 18 months. | " | | | | Laserre. | Recovered in four weeks. |
| 42 | 1835 | | " | " | | " | | | | " | | | | Cook. | Ligation followed by amputation in three weeks. |
| 43 | 1836 | | " | " | | | | | | " | | | | Mooz. | |
| 44 | 1840 | | " | " | | " | | | 4 years. | " | | | | Portal. | Recovered in 29 days. |
| 45 | 1842 | | " | " | | | | " | | " | | " | | Betschler. | |
| 46 | 1842 | | " | " | | | " | | | " | | " | | Juergenes. | Ligated with silver wire, then amputated. |
| 47 | 1843 | | " | " | | " | | | 1 month. | " | | | | Crosse. | Recovered in four weeks. |
| 48 | 1848 | | " | " | | " | | | 5 years. | " | | | | Johnson. | Recovered in six weeks. |
| 49 | 1848 | | " | " | | " | | | 2 months. | " | | | | Hublier. | |
| 50 | 1849 | | " | " | | " | | | 2 years. | " | | | | Higgins. | Quick recovery. |
| 51 | 1854 | | " | " | | | | " | | " | | " | | Gredding. | |
| 52 | 1859 | | " | " | | " | | | | " | | | | McClintock. | Ecraseur applied after the ligature. |
| 53 | 1863 | | " | " | | " | | | | " | | | | Sheppard. | |
| 54 | 1803 | | " | " | | | | | | | " | | | Watkinson. | Ligature slid off, causing fatal hemorrhage. |
| 55 | 1836 | | " | " | | " | | | 1 year. | | " | | | Meirholdt. | Internal hemorrhage, peritonitis and death in 19 days. |
| 56 | 1840 | | " | " | | " | | | | | " | | | Velpeau. | Death in 72 hours. |
| 57 | 1850 | | " | " | | | " | | 3 years. | | " | " | | Engel. | Death in 7 days. |
| 58 | 1867 | | " | " | | | | " | | | " | " | | Scanzoni. | |
| 59 | 18— | | | | " | " | | | 8 hours. | " | | | " | Channing. | Case not diagnosed by attending physician at the time. |
| 60 | 18— | | | | " | " | | | 10 hours. | " | | " | | Channing. | Reduced in a few minutes. |
| 61 | 18— | 24 | " | | | " | | | 18 months. | " | | | " | Channing. | Placenta torn away in pieces. |
| 62 | 18— | 22 | " | | | " | | | 12 months. | " | | | " | Channing. | Ligature came away in 13 days. |
| 63 | 18— | 35 | " | | | " | | | 12 months. | | " | " | | Channing. | Not diagnosed by attending physician. |
| 64 | 18— | | " | | | " | | | | " | | | " | Channing. | |
| 65 | 18— | | " | | | " | | | | " | | " | | Channing. | Not diagnosed by attending physician at the time. |
| 66 | 18— | | " | | | " | | | | " | | | " | Channing. | Mistaken for polypus. |
| 67 | 18— | | " | | | " | | | | " | | | " | Channing. | Mistaken for polypus. |
| 68 | 18— | 22 | " | | | " | | | 18 months. | " | | " | | Channing. | Reduction attempted before ligation. |
| 69 | 1838 | 20 | | | | " | | | 2 months. | | | " | | Fisher. | Patient would not consent to reduction. |
| 70 | | | | | " | " | | | 1 hour. | " | | | " | Fisher. | Reduced before the placenta was removed. |
| 71 | 1837 | | | | " | " | | | 1 hour. | | " | | " | Richards | Died in six hours. |
| 72 | 1867 | | | | " | " | | | 1 hour. | " | | " | | Warren. | Five and a half months advanced in pregnancy. |
| 73 | 1859 | 21 | | | " | " | | | 6 months. | " | | " | | Potter. | Time, one and a half hours in reduction. |
| 74 | 1840 | 30 | | | " | " | | | 15 months. | " | | | " | Potter. | Reduced in three quarters of an hour |
| 75 | 1836 | 35 | | | " | " | | | 6 months. | " | | " | | Potter. | First mistaken for polypus; reduced in three minutes. |
| 76 | 1870 | 12 | | | " | " | | | ¼ hour. | " | | | " | Cowards. | Reduced in twenty minutes. |
| 77 | | | | | " | " | | | ¼ hour. | " | | | " | Madge. | Placenta peeled off before reduction. |
| 78 | | | | | " | " | | | 1-6 hour. | " | | " | | Hicks. | |
| 79 | | | | | " | " | | | 1-6 hour. | " | | " | | Hicks. | |
| 80 | 1868 | | | | " | " | | | 5 hours. | " | | " | | Tylecote. | |
| 81 | 1871 | 22 | | | " | " | | | 54 hours. | | " | " | | Woodward. | |
| 82 | 1870 | 22 | | | " | " | | | 2 months. | " | | " | | Byford. | Inversion took place 35 days after confinement and was reduced by elastic pressure with water in five days. |
| 83 | 1860 | 26 | | | " | " | | | soon. | " | | " | | Hamill. | Inversion took place immediately after birth of child. |
| 84 | 1868 | 28 | | | " | " | | | soon. | " | | " | | Calkins. | Inversion took place immediately after birth of child. |
| 85 | 1868 | 22 | | | " | " | | | 52 hours. | " | | | " | Holmes. | Homœopathic physician in attendance. |
| 86 | 1868 | | | | " | " | | | 25 days. | " | | " | | Trist. | First mistaken for polypus, after which spontaneous reduction took place. |
| 87 | 1866 | | | | " | " | | | soon. | " | | " | | Sidey. | Reduced immediately after accident. |
| 88 | | 19 | | | " | " | | | soon. | " | | | " | Denham. | The only case in 100,000 deliveries in Dublin Lying-in Hospital. |

| Number. | Years. | Age. | Mode of operating. Ligation. | Amputation. | Reduction. | Causes. Post Partum. | Polypus. | Fibroid. | Time after Accident | Results of treatment. Successful. | Unsuccessful. | Kinds. Spontaneous. | Artificial. | Authority. | Remarks. |
|---|---|---|---|---|---|---|---|---|---|---|---|---|---|---|---|
| 89 | 1866 | 33 | | | " | " | | | 15 days. | " | | | " | Denham. | Not diagnosed for 11 days. |
| 90 | 1866 | 27 | | | " | " | | | 4 years. | " | | " | | Woster. | Reduction completed after 8 attempts of 9 hours' duration; the last of 3 hours. |
| 91 | 1865 | 24 | | | " | " | | | 7 months. | " | | " | | Emmett. | Time in reduction, 5 hours 55 min. |
| 92 | 1862 | | | | " | " | | | 13 years. | " | | | " | Noeggorath. | Operation by pressing one horn of uterus. |
| 93 | | | | | " | " | | | | " | | | " | Noeggorath. | One side of uterus pressed in first. |
| 94 | 1866 | | | | " | " | | | 8 months. | " | | | " | Emmett. | Reduced in 1 hour 20 minutes. |
| 95 | 1864 | | | | " | " | | | 2 hours. | | " | " | | Smith and Cutter. | Death from shock. |
| 96 | 1869 | | | | " | | | " | 2 years. | " | | " | | Emmett. | The fundus pushed into the os and sutures applied to hold it there without completing the reduction. |
| 97 | 1868 | | | | " | " | | | | " | | " | | Emmett. | This case was completed after several hours manipulation by sutures in the os to hold up the fundus. |
| 98 | | | | | " | " | | | 8 months. | " | | | " | Debourgeville | |
| 99 | | | | | " | " | | | 8 years. | " | | | " | Baudelœque. | Reduced in ninety minutes. |
| 100 | 1847 | | | | " | " | | | 1 year. | " | | | " | Valentine. | Reduced in ten minutes. |
| 101 | 1852 | | | | " | " | | | 5 months. | " | | | " | Canner. | |
| 102 | 1852 | | | | " | " | | | 13 months. | " | | | " | Berrier. | |
| 103 | 1856 | | | | " | " | | | 12 years. | " | | | " | Tyler Smith. | Elastic pressure with a caoutchouc bag filled with air, in one week. |
| 104 | 1858 | | | | " | " | | | 16 years. | | " | | " | J. P. White. | The celebrated case of Dr. White—reduced in 50 minutes; patient died of peritonitis 15 days after the operation. |
| 105 | 1869 | 28 | | | " | " | | | 10 months. | " | | " | | Thomas. | Not diagnosed for nine months; belladonna suppositories used for 3 days, then reduction accomplished in 25 minutes. |
| 106 | 1869 | 23 | | | " | " | | | 21 months. | " | | " | | Thomas. | Preparatory treatment same as last; taxis 31 hours without result; then abdominal walls incised, os uteri dilated from above, taxis below. |
| 107 | 1860 | | | | " | " | | | soon. | " | | " | | Tanner. | Inversion with placenta attached; separated and reduced. |
| 108 | 1860 | | | | " | " | | | soon. | " | | " | | Tanner. | Placenta attached; separated and reduced. |
| 109 | 1860 | | | | " | " | | | soon. | | " | " | | Fifield. | Placenta attached; separated before reduction; died in a few hours. |
| 110 | 1866 | | | | " | " | | | 21 hours. | " | | " | | Emmett. | Displacement came on after the attending physician left the house. |
| 111 | 1867 | | | | " | | | | 2 months. | " | | " | | Emmett. | Unimpregnated uterus, and without other assignable cause. |
| 112 | 1871 | 27 | | | " | " | | | 5 weeks. | " | | | " | White. | Reduced in 23 minutes. |
| 113 | 1871 | 34 | | | " | " | | | 6 months. | " | | | " | White. | Reduced in 1 hour 23 minutes by taxis, with uterine repository. |

*Case.*—I was called to see Mrs. W., in an adjoining town, on the morning of Jan. 30, 1872, and reached the address toward noon. Her medical attendant was in waiting for me, from whom I obtained the following history:

Mrs. W., aged 25, was delivered of her first child two weeks previous; labor natural and easy, lasting about ten hours. Not so with the after-birth, as excessive flowing accompanied and followed its removal, in so much that the patient came near dying from nervous shock, and fatal syncope from loss of blood. The placenta having come away prior to, or immediately at the time of the inversion, the latter was not detected.

The patient lay in a very critical condition for a number of days, fainting every time the head was raised from the pillow. A neighboring physician was called in, and supposing the symptoms were in consequence of the loss of blood that had been going on more or less profusely since her confinement, did not make an examination, and up to the time I saw the patient the case had not been diagnosed. The attending physician said that no undue traction upon the cord had been used in the delivery of the placenta, but could not say that he had felt the uterus above the symphysis at any time after the completion of the accouchment.

In the morning before I saw the patient, she stepped upon the floor for the first time, when the uterus immediately protruded through the vulva. This so alarmed the friends that I was sent for. Meantime the attending physician pressed the uterus into the vagina, applied a compress, and supposed the difficulty entirely relieved. I found the patient upon the bed, presenting every indication of extreme exhaustion from anæmia, with a pulse of 160 per minute. On removing the compress and making a vaginal examination, a large firm mass, about three and a-half inches in length by two and a-half in breadth, was felt blocking the vagina, and might as well have been mistaken for polypus as it already had been for prolapsus. I passed two fingers around the projecting mass, and found the continuity perfect. I then manipulated with the other hand above the symphysis pubis, and was able to so far approximate the one in the cul-de-sac, as to sufficiently satisfy myself that the case was one of inversion of the uterus. As the patient and friends had been informed, when the uterus was slipped into the vagina, that "everything was right," it was with much difficulty that I succeeded in satisfying their minds in regard to the facts in the case, and the necessity of immediately proceeding to the use of taxis for the purpose of reducing the inverted organ, as it was a serious question in my mind whether anything could be gained by delay, and everything might be lost.

The patient was placed upon her back, with her knees drawn up, and in such position that I could operate while sitting or standing. The right hand was passed into the vagina, the uterus grasped and pressed firmly between the fingers, while at the same time an upward pressure of from fifteen to twenty pounds was made against the left hand, which was placed upon the abdomen for the purpose of counter pressure. This pressure was continued about five minutes each time, with an interval of the same length of time between. At the end of twenty minutes I found the neck materially dilated, and the point of constriction rapidly giving way upon each application of the force. I then made the pressure more continuous, and the interval shorter each time, for the next ten minutes, when the reduction was completed, not with a bound, but requiring about the same amount of force to the very last. By this time the patient was very much exhausted, but rallied speedily under the influence of stimulants and small doses of morphine, together with beef essence. She was kept quiet, in a recumbent position, for the next two weeks, with no other treatment save an occasional dose of morphine, to relieve pain and tenderness over the abdomen.

